# Transmission of SARS‐CoV‐2 during a 2‐h domestic flight to Okinawa, Japan, March 2020

**DOI:** 10.1111/irv.12913

**Published:** 2021-10-03

**Authors:** Takao Toyokawa, Tomoe Shimada, Takahiro Hayamizu, Tsuyoshi Sekizuka, Yuji Zukeyama, Miyako Yasuda, Yuko Nakamura, Sho Okano, Jun Kudaka, Tetsuya Kakita, Makoto Kuroda, Tadashi Nakasone

**Affiliations:** ^1^ COVID‐19 Response Team Naha City Public Health Center Naha‐shi Okinawa Japan; ^2^ Infectious Disease Surveillance Center National Institute of Infectious Diseases (NIID) Shinjuku‐ku Tokyo Japan; ^3^ Pathogen Genomics Center National Institute of Infectious Diseases (NIID) Shinjuku‐ku Tokyo Japan; ^4^ Regional Health Division, Department of Public Health and Medical Care Okinawa Prefectural Government Naha‐shi Okinawa Japan; ^5^ Okinawa Prefectural Institute of Health and Environment Uruma‐shi Okinawa Japan

**Keywords:** cohort studies, COVID‐19, disease outbreaks, molecular epidemiology, SARS‐CoV‐2, travel‐related illness

## Abstract

**Background:**

Coronavirus disease (COVID‐19), caused by severe acute respiratory syndrome coronavirus (SARS‐CoV‐2), has rapidly spread globally. Potentially infected individuals travel on commercial aircraft. Thus, this study aimed to investigate and test the association between the use of face masks, physical distance, and COVID‐19 among passengers and flight attendants exposed to a COVID‐19 passenger in a domestic flight.

**Methods:**

This observational study investigated passengers and flight attendants exposed to COVID‐19 on March 23, 2020, on board a flight to Naha City, Japan. Secondary attack rates were calculated. Whole‐genome sequencing of SARS‐CoV‐2 was used to identify the infectious linkage between confirmed cases in this clustering. The association between confirmed COVID‐19 and proximity of passengers' seats to the index case and/or the use of face masks was estimated using logistic regression.

**Results:**

Fourteen confirmed and six probable cases were identified among passengers and flight attendants. The secondary attack rate was 9.7%. Twelve of 14 SARS‐CoV‐2 genome sequences in confirmed cases were identical to that of the index case or showed only one nucleotide mutation. Risk factors for infection included not using a face mask (adjusted odds ratio [aOR]: 7.29, 95% confidence interval [95% CI]: 1.86‐28.6), partial face mask use (aOR: 3.0, 95% CI: 0.83‐10.8), and being seated within two rows from the index patient (aOR: 7.47, 95% CI: 2.06‐27.2).

**Conclusion:**

SARS‐CoV‐2 was transmitted on the airplane. Nonuse of face masks was identified as an independent risk factor for contracting COVID‐19 on the airplane.

## 1 INTRODUCTION

The risk of in‐flight transmission of severe acute respiratory syndrome coronavirus 2 (SARS‐CoV‐2) infection has been reported to be low.^1^ Well‐documented cases of SARS‐CoV‐2 transmission in airplanes have mostly occurred on international flights,^2^, ^3^, ^4^, ^5^, ^6^, ^7^, ^8^ thus suggesting a low risk of infection on domestic flights, which have relatively short flight times.^9^ Given that physical distancing is impossible during flight travel, other measures such as the use of face masks play an important role in preventing viral transmission. Recent reports^1^, ^10^ have suggested that face mask use effectively reduces the risk of SARS‐CoV‐2 infection; however, few reports indicate association between the precautionary measures and infection incidence. We investigated a cluster of coronavirus disease (COVID‐19) cases among travelers who were likely exposed to an index patient in an airplane and tested the associated risk of infection according to face mask use and passengers' seats proximity to that of the index patient.

### 1.1 Outbreak detection

On March 26, 2020, a health center in Okinawa Prefecture was notified by a physician that a man in his 30s, a resident of Naha, Japan, had been diagnosed with COVID‐19 based on positive SARS‐CoV‐2 polymerase chain reaction (PCR) test results. The cycle threshold (Ct) value of his nasopharyngeal swab sample collected on March 24 was 18.8. He had traveled to Prefecture A in Kansai on the mainland of Japan on March 20. He had developed a fever and cough on March 23. Despite being symptomatic, he had returned to Naha via a 2‐h domestic, commercial airline flight from City X in Prefecture B on March 23, without wearing a face mask. We investigated the passengers and flight attendants for SARS‐CoV‐2 infection and discovered a cluster of cases. This report describes the investigation of SARS‐CoV‐2 infection clustering among the passengers and crew on the flight on March 23 and the association between face mask use, physical distancing, and the risk of acquiring SARS‐CoV‐2 infection.

## 2 METHODS

This cohort study investigated passengers and flight attendants who boarded the flight at the airport in Prefecture B and traveled to Naha Airport in Okinawa on March 23, 2020.

### 2.1 Setting

The airplane was a Boeing 737‐800 with 177 economy‐class seats. It had an air recirculation system equipped with high‐efficiency particulate air (HEPA) filtration. We confirmed that the HEPA filter of this airplane had been changed on February 15, 2020. The airplane was boarded by 148 persons: the index patient, 141 other passengers (seat occupancy: 80.2%), four flight attendants, and two pilots. We verified with the airline office that there was no flight delay and that passengers and crew members boarded and disembarked the plane using the boarding bridge.

### 2.2 Investigation of the index case, passengers, and flight attendants

With the physician's assistance, we investigated the index patient over the phone. We obtained information, including name, age, sex, seat assignment, and telephone number of the patient's fellow passengers, from the passenger manifest.

**FIGURE 1:**
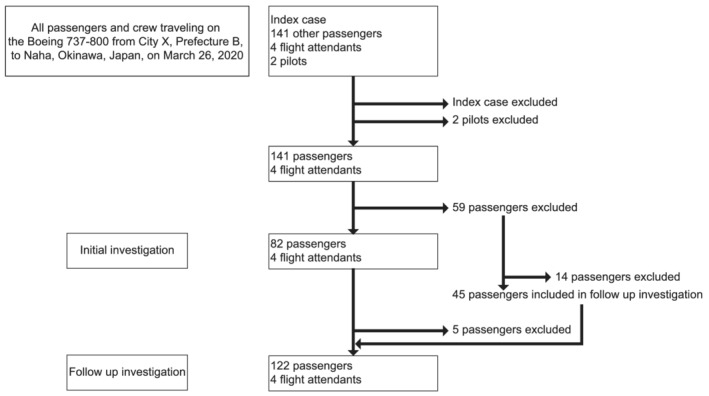
Flowchart showing the selection process of the study subjects on board the airplane with the index case of coronavirus disease

At the beginning of our investigation, we traced the passengers who sat within two rows from the index patient. However, because symptomatic passengers with positive PCR test results were reported by the health department of another prefecture, we expanded our contact tracing as shown in Figure 1: initial investigation and follow‐up. In the initial investigation, we interviewed 82 out of all 141 passengers between March 26 and April 6, 2020. The passengers were notified by telephone about their potential exposure to SARS‐CoV‐2 infection and were asked to self‐quarantine and self‐monitor until April 6, which was 14 days after the flight travel. We advised them to consult their local COVID‐19 consultation center if they experienced any symptoms. We requested the airline to inform the flight attendants in the same manner.

### 2.3 Follow‐up investigation

To investigate and test the association between mask wearing and physical distancing with COVID‐19 incidence in passengers and flight attendants exposed to a passenger with COVID‐19 on a domestic flight, we carried out the follow‐up investigation during April 26–29, 2020. We developed a validated questionnaire and obtained information via a telephone interview. We interviewed 122 out of the 141 passengers, including the 77 who had been interviewed earlier and 45 who had not been contacted in the initial investigation, on their characteristics, seat assignment, face mask use during the flight travel (Always; Most of the time; Never/did not have a face mask; Cannot remember; Others), and symptoms of illness (fever ≥37.5°C, cough, nasal discharge/obstruction, sore throat, shortness of breath/difficulty breathing, fatigue, nausea/vomiting, headache, myalgia/arthralgia, hyposmia/hypogeusia) between March 23 and April 6, 2020. We requested the airline to administer the questionnaire to the flight attendants and received the completed forms by email. Thus, we followed up a total of 126 participants for 34 days after the index case was reported.

Additionally, we requested the local health authorities and public health centers in seven prefectures to share the data of confirmed cases in each epidemiological investigation and send each specimen residue used for diagnosis to the Pathogen Genomics Center, National Institute of Infectious Diseases, Japan.

### 2.4 Ethical considerations

This study was conducted as an active epidemiological investigation under Article 15 of the Japanese Infectious Disease Law in cooperation with local health authorities, public health centers, and public health institutes. Thus, the study was exempt from the institutional review board review, and informed consent was not required.

### 2.5 Case definition

We defined a confirmed case as an instance in which a passenger or flight attendant on board had SARS‐CoV‐2 infection confirmed between March 23 and April 6, 2020, regardless of whether symptoms or signs developed. We defined a probable case as an instance in which a passenger or flight attendant on board this flight had an acute respiratory illness (fever ≥37.5°C and at least one sign/symptom of respiratory disease, e.g., cough, sore throat, nasal discharge/obstruction, and shortness of breath) but did not undergo PCR testing between March 23 and April 6, 2020. The day of symptom onset was defined as the day on which the first symptom occurred in confirmed or probable cases based on epidemiological data. Secondary attack rates were calculated as the number of confirmed cases divided by the number of passengers and flight attendants, excluding the index case.

### 2.6 Laboratory analysis

Nasopharyngeal swab specimens were collected from patients, and real‐time reverse‐transcription quantitative PCR (RT‐qPCR) testing for SARS‐CoV‐2^11^ was performed. An epidemiological investigation was performed using positive RNA samples subjected to whole‐genome sequencing according to the PrimalSeq protocol for enriching complementary DNA of the SARS‐CoV‐2 genome using multiplex RT‐PCR,^12^ followed by discriminating the linkage between confirmed cases in this clustering based on genome‐wide single nucleotide variations.

### 2.7 Statistical analysis

Confirmed or probable cases were identified as outcome variables. Univariate and multivariate logistic regression analyses were performed, respectively, to calculate the crude and adjusted relative risk of COVID‐19 among passengers seated within two rows from the index case or those wearing masks. We evaluated the proximity of a passenger's seat to the index case according to the World Health Organization guideline for close contact with COVID‐19 in travel‐associated settings, which is whether a passenger was seated within two rows of the index patient. The analysis included 121 passengers and four flight attendants from whom information could be obtained in the follow‐up investigation in addition to the index patient. Regarding face mask use, no respondent selected “Others,” and only one passenger selected “Cannot remember.” Therefore, we used three categories, namely, “Always,” “Most of the time,” and “Never/did not have a face mask” as independent variables in the logistic regression analysis. All analyses were performed using SAS, Version 9.4 (SAS Institute Inc., Cary, NC, USA).

## 3 RESULTS

**FIGURE 2:**
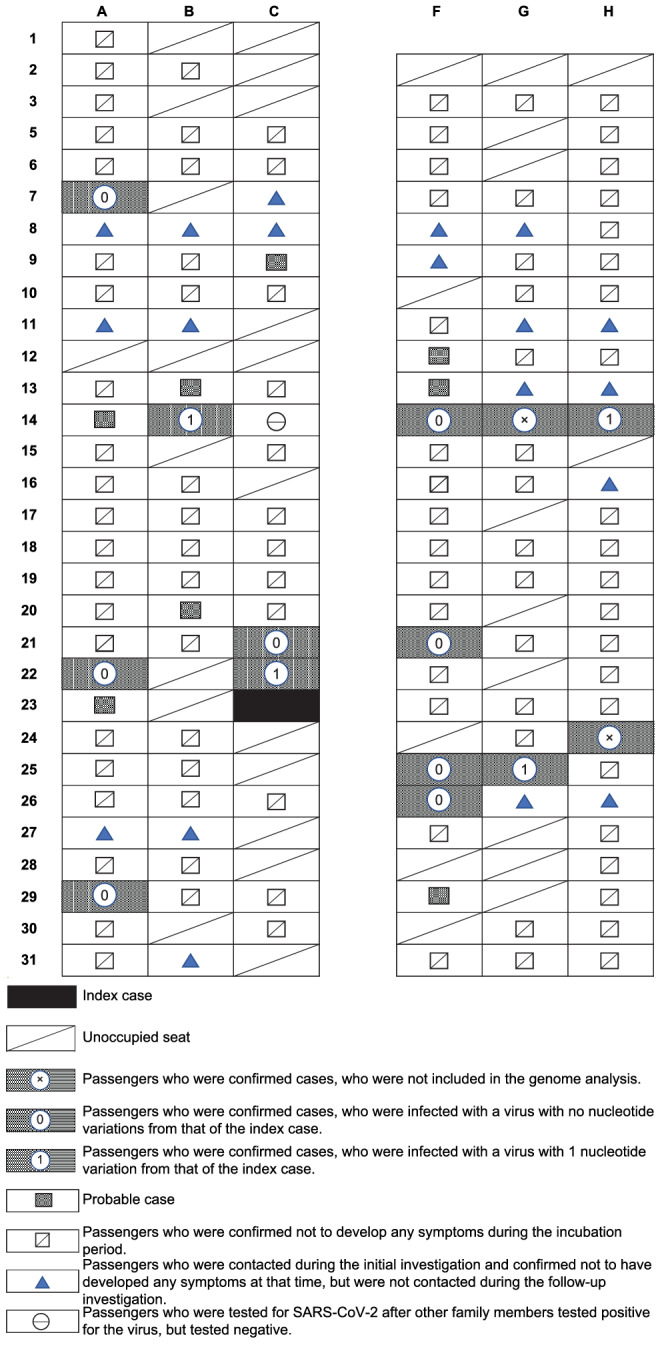
Schematic diagram of the seat assignments of passengers traveling on Boeing 737‐800 from City X, Prefecture B, to Naha, Okinawa, Japan, on March 23, 2020. SNV, single nucleotide variation

**FIGURE 3:**
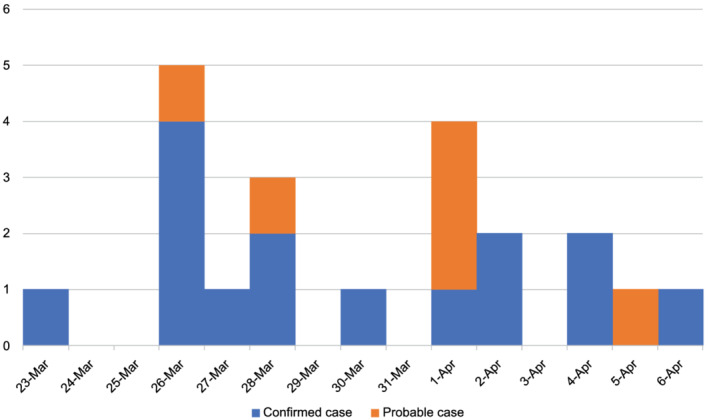
Day of symptom onset for confirmed and probable cases of coronavirus disease (COVID‐19) among passengers of Boeing 737–800 from City X, Prefecture B, to Naha, Okinawa, Japan, on March 23, 2020 (*N* = 21)

The 146 individuals aboard the flight (excluding the two pilots who did not share the cabin space during the flight) had a median age of 26 years (range, 3–85 years), and 74 (50.7%) of them were male. Among these individuals, 14 confirmed cases and six probable cases were identified (Figures 2 and 3; Table 1).

**TABLE 1:** Timeline of the onset of illness and diagnosis of 15 confirmed cases of COVID‐19 transmitted on the flight to Naha, Okinawa, Japan, on March 23, 2020 (*N* = 15)

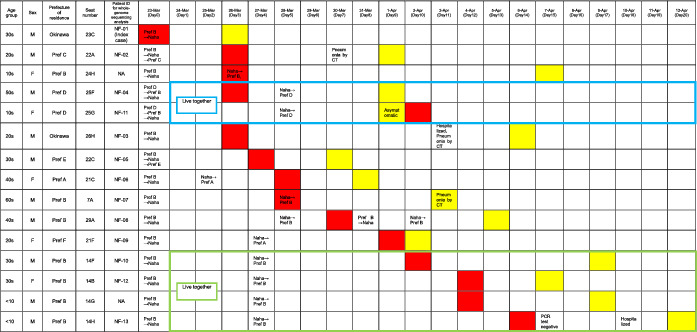

*Notes*: For each case, the day of symptom onset is shown in red, and the day of testing positive for SARS‐CoV‐2 using reverse‐transcription polymerase chain reaction is shown in yellow. The passengers in seats 25F and 25G lived together (blue box), and the passengers in seats 14B, 14F, 14G, and 14H lived together (green box).

Abbreviations: COVID‐19, coronavirus disease; NA, not available; SARS‐CoV‐2, severe acute respiratory syndrome coronavirus 2.

Additionally, based on an epidemiological investigation conducted by each municipality, none of the 14 individuals with confirmed COVID‐19 were identified as having a history of close contact with a confirmed COVID‐19 patient other than the index patient and other confirmed cases on the flight. Furthermore, we were able to determine the genome sequence of the virus in 12 of the 14 confirmed cases that were all either identical to that in the index case— classified as the progeny of the Europe‐related Japanese isolates (Pangolin lineage B.1.1)^13^—or differed by only one nucleotide (Table 2). In‐flight exposure is thus the most likely explanation for this outbreak.

**TABLE 2:** Summary of the single nucleotide variation alleles of SARS‐CoV‐2 isolates in this study

	Wuhan‐Hu‐1 genome position (MN908947.3)	241	313	3037	5011	14 209	14 408	23 403	25 424	26 730	28 087	28 881	28 882	28 883	
		C	C	C	A	G	C	A	G	G	C	G	G	G	
Patient ID	Variations to index case (NF‐01)														GISAID isolate ID
NF‐01	‐	T	T	T	A	G	T	G	G	A	C	A	A	C	EPI_ISL_591471
NF‐02	0	T	T	T	A	G	T	G	G	A	C	A	A	C	EPI_ISL_591472
NF‐03	0	T	T	T	A	G	T	G	G	A	C	A	A	C	EPI_ISL_591473
NF‐04	0	T	T	T	A	G	T	G	G	A	C	A	A	C	EPI_ISL_591474
NF‐05	1	T	T	T	A	T	T	G	G	A	C	A	A	C	EPI_ISL_591475
NF‐06	0	T	T	T	A	G	T	G	G	A	C	A	A	C	EPI_ISL_591476
NF‐07	0	T	T	T	A	G	T	G	G	A	C	A	A	C	EPI_ISL_591477
NF‐08	0	T	T	T	A	G	T	G	G	A	C	A	A	C	EPI_ISL_591478
NF‐09	0	T	T	T	A	G	T	G	G	A	C	A	A	C	EPI_ISL_591479
NF‐10	0	T	T	T	A	G	T	G	G	A	C	A	A	C	EPI_ISL_591480
NF‐11	1	T	T	T	A	G	T	G	G	A	T	A	A	C	EPI_ISL_591481
NF‐12	1	T	T	T	A	G	T	G	T	A	C	A	A	C	EPI_ISL_591482
NF‐13	1	T	T	T	C	G	T	G	G	A	C	A	A	C	EPI_ISL_591483

Abbreviation: GISAID, Global Initiative on Sharing All Influenza Data.

There were two family clusters among the 14 confirmed cases, seated in 14B, 14F, 14G, and 14H and 25F and 25G. Given the incubation period and the results of the whole‐genome sequencing, two scenarios are possible: either two family members (14B, 14F) were directly infected by the index patient or the passenger seated in 14F, or the first family member to develop COVID‐19 was directly infected by the index patient and then transmitted the infection to the patient seated in 14B. Similarly, passengers seated in 25F and 25G might have both been directly infected by the index patient. Alternatively, the passenger seated in 25F, who was the first family member to develop COVID‐19, may have transmitted the infection to the passenger seated in 25G. The secondary attack rate was 9.7%. If probable cases were included, the attack rate would be 13.8%. Regarding confirmed cases in the seven prefectures where the 14 patients with confirmed COVID‐19 had lived during the period March 1–23, 2020, a cumulative total of 20–130 cases were reported in three prefectures, three cases in one prefecture, and no suspected cases were reported in the other three prefectures, including Okinawa Prefecture.

As shown in Table 3, the relative risks and 95% confidence intervals (CIs) of inappropriate use or nonuse of face masks and passengers seated within two rows from the index patient were 2.46 (95% CI: 0.75–8.09), 4.6 (95% CI: 1.28–16.6), and 4.8 (95% CI: 1.46–15.8), respectively. Nonuse of face mask was identified as an independent risk factor after adjusting for being seated within the two rows from the index patient.

**TABLE 3:** Association of the use of face mask and maintaining distance from the index patient with SARS‐CoV‐2 positivity

Variables	Number of passengers and flight attendants	Number of cases^a^ (%)	Crude OR		Adjusted^b^ OR	
			OR	95% CI	OR	95% CI
Wore a mask all the time	92	11 (12)	1.00		1.00	
Wore a mask most of the time	20	5 (25)	2.46	0.75–8.09	3.00	0.83–10.8
Did not wear a mask	13	5 (38)	4.60	1.28–16.6	7.29	1.86–28.6
Seat beyond two rows from the index patient	111	15 (14)	1.00		1.00	
Seat within two rows from the index patient	14	6 (43)	4.80	1.46–15.8	7.47	2.06–27.2

Abbreviations: CI, confidence interval; OR, odds ratio.

^a^ Confirmed and probable cases.

^b^ Adjusted for wearing a face mask and maintaining distance from the index case, simultaneously.

## 4 DISCUSSION

Our investigation results suggest that SARS‐CoV‐2 was transmitted from the index patient to others during the 2‐h flight. Combined epidemiological‐genomics analyses suggested the possibility that in‐flight transmission occurred through infected respiratory droplets and infected micro‐aerosols. Moreover, our report confirms the effectiveness of using face masks among passengers and flight attendants during commercial air travel.

The potential for in‐flight transmission of infection, especially aerosol transmission, is low^14^, ^15^, ^16^ because HEPA filters are used in aircraft ventilation systems. The so‐called two‐row rule, which designates passengers seated within two rows from a confirmed patient as concentrated contacts owing to the risk of droplet infection, is internationally applied to identify contacts of infected airplane passengers.^17^ However, transmission—including SARS and Middle East respiratory syndrome viruses, which are members of the family of coronaviruses that infect humans—is possible not only through infected droplets but also through aerosols.^18^, ^19^ Recent reports indicate a potential risk of airborne transmission of SARS‐CoV‐2, and the US Centers for Disease Control and Prevention (CDC) updated its guidelines on SARS‐CoV‐2 spread through airborne transmission, wherein droplet transmission alone cannot explain the spread.^20^, ^21^, ^22^, ^23^, ^24^ The fact that most passengers with COVID‐19 on the flight were seated more than two rows away from the index case raises the possibility that this cluster was attributed to infected micro‐aerosols to a certain extent.

The estimated basic reproductive number (R_0_) for COVID‐19 from China at the beginning of the pandemic was approximately 2.2–2.7.^25^, ^26^, ^27^ However, recent studies have shown that individuals can cause many secondary infection cases, especially under special conditions, including closed spaces, crowded places, and close‐contact settings.^28^, ^29^, ^30^ The cabin environment of a commercial airplane is essentially crowded and enclosed, making it conducive to disease spread through infected aerosols.^31^ Our findings suggest that contact tracing for airplane events might be necessary to move beyond the scope of conventional rules and in some cases investigate all passengers and flight attendants. In this example, the clustering occurred on a relatively short‐distance domestic flight (2‐h travel). Some contributory factors were noted: the date of symptom onset of the index patient, which is thought to be the period of high viral shedding^32^, ^33^; the high viral load of the index patient's specimen, which was collected on the day after the flight; and the fact that the index patient was not wearing a face mask during the flight, despite his intense cough, which is a common source of aerosols.^31^ Additionally, in terms of airflow in the cabin, the aisle seats, such as the index patient's seat, are located upstream of cabin airflow, according to the International Air Transport Association (IATA), which could have led to the diffusion of aerosols into the cabin space.^34^ These factors might have contributed to this clustering. Nonetheless, despite these factors, the relatively low secondary attack rate may indicate that the cabin ventilation system worked effectively. Simultaneously, when conducting a risk assessment of an index patient on an airplane, a two‐tiered approach can be followed: first, the two‐row rule as the initial step, and second, if more than one person is positive or suspected of being infected, the investigation should be extended to all passengers.

Many countries, including Japan, have imposed travel restrictions and mandatory quarantine requirements to control the spread of COVID‐19. We need to consider ways to lower the risk of clusters of secondary infections transmitted during air travel and mitigate its impact.

To reduce the risk of passengers being infected with COVID‐19, a screening system is required, such as a temperature check before boarding, which some countries have already implemented, and screening of passengers at airports for the presence of COVID‐19 symptoms. To address the risk of asymptomatic travelers, it may be worth screening passengers for potential exposure or contact with confirmed COVID‐19 patients or travel history to a COVID‐19 epidemic area in the past 14 days, and performing PCR or rapid antigen testing before boarding, even on a domestic flight, depending on the passenger's risk. Even if all passengers are required to take a PCR test before boarding as a screening test, it is impossible to detect all infected individuals due to the test's limitations. One case was reported in which the passenger with a negative PCR test before boarding was thought to be the source of the in‐flight transmission of infection.^8^ Therefore, it is essential to take countermeasures in case an infected person board the plane. Specifically, it is necessary to minimize and manage the outbreak impact; a vigorous investigation system must be developed by the health authority. Even if various proactive pre‐boarding measures were implemented, transmission from an asymptomatic person or pre‐onset transmission could occur. For example, although active epidemiological investigations of airplane passengers may cross many municipalities and sometimes national borders, the median incubation period of the disease is short (4–5 days)^35^ and requires a rapid response. Measures should include guidelines for sharing information from the passenger manifests rapidly between the responsible health authority and the airline company. The same information should be shared with other health authorities of regions where the exposed passengers or flight attendants reside, nationally and internationally. Information technology, which can facilitate disease tracking of passengers and can share information across national borders, could also play an important role.

Additionally, a universal mask‐wearing policy in the plane must be discussed in this COVID‐19 era. Using a face mask during flight travel effectively decreased the risk of acquiring SARS‐CoV‐2 infection in our study. Some recent studies have suggested that wearing masks is beneficial not only to prevent the spread of the virus from infected people to others but also to provide personal protection.^36^, ^37^, ^38^ As IATA pointed out,^9^ most reports published to date^2^, ^3^, ^4^, ^5^, ^6^, ^7^, ^8^ and our study indicated the potential for in‐flight transmission of COVID‐19 in the early stages of the epidemic, before control measures, such as masks wearing, were implemented.

This study has some limitations. First, passengers' information was obtained via telephone interview, and recall bias cannot be excluded. Second, we could not investigate all passengers and evaluate all asymptomatic passengers for SARS‐CoV‐2 infection using an antibody test; thus, we may have missed some passengers with COVID‐19 and underestimated the impact of this event. Third, 19 passengers were lost to follow‐up; however, we believe that the loss was randomly distributed among the passengers and was unrelated to their disease status. Therefore, loss to follow‐up did not have an epidemiologically important impact on our findings. Fourth, we may have overestimated the attack rate by counting confirmed cases that may have been transmitted within the family at home rather than in the flight and assuming that all passengers classified as probable cases were infected with SARS‐CoV‐2. As their infection status was not confirmed on RT‐PCR testing, we cannot exclude the possibility that their symptoms were due to other conditions or diseases, especially influenza. However, this is unlikely because according to the National Epidemiological Surveillance of Infectious Diseases system, the incidence of influenza was below 10.0 cases/sentinel/week at the time—below the threshold for an alert. Fifth, those infected with SARS‐CoV‐2 might not have been infected while they were seated in the cabin of the airplane. Activities during their travel, their close contacts, or contaminated surfaces of seatback or in the aisle or toilets of the airplane, the baggage claim, and departures lobby or lounge in the airport were potential opportunities for exposure to the index patient. Finally, we did not evaluate how well the ventilation system functioned in the aircraft at the time of this event.

Our study shows that aerosol‐based transmission of SARS‐CoV‐2 may occur during a short domestic flight. Wearing face masks during flight travel was associated with a reduced risk of SARS‐CoV‐2 infection. Now that the spread of variants of SARS‐CoV‐2 across regions and countries has become a global threat, awareness on the use of face masks, an effective pre‐boarding screening system, and a vigorous investigation system are necessary to minimize the risk of in‐flight viral transmission. Additionally, this cluster occurred in March 2020 before the emergence of several variants of SARS‐CoV‐2 with confirmed increased transmissibility. In the future, it will be necessary to assess the risk of in‐flight transmission of these variants.

## AUTHOR CONTRIBUTIONS

**Takao Toyokawa:** Conceptualization; data curation; formal analysis; investigation; methodology; resources; software; supervision; validation; visualization. **Tomoe Shimada:** Conceptualization; data curation; formal analysis; funding acquisition; methodology; software; supervision; validation; visualization. **Takahiro Hayamizu:** Data curation; investigation; methodology; resources. **Tsuyoshi Sekizuka:** Data curation; formal analysis; investigation; methodology; software; validation; visualization. **Yuji Zukeyama:** Data curation; investigation. **Miyako Yasuda:** Data curation; investigation. **Yuko Nakamura:** Data curation; investigation. **Sho Okano:** Data curation; investigation. **Jun Kudaka:** Data curation; investigation. **Tetsuya Kakita:** Data curation; investigation. **Makoto Kuroda:** Data curation; formal analysis; funding acquisition; investigation; methodology; resources; software; validation; visualization. **Tadashi Nakasone:** Conceptualization; investigation; project administration; resources; supervision.

## CONFLICT OF INTEREST

None declared.

### PEER REVIEW

The peer review history for this article is available at https://publons.com/publon/10.1111/irv.12913.

## Data Availability

The new sequences have been deposited in the Global Initiative on Sharing All Influenza Data (GISAID) with accession IDs EPI_ISL_591471 to EPI_ISL_ 591 483 (Table 2).
